# Systemic immune-inflammation index (SII): A More Promising Inflammation-Based Prognostic Marker for Patients with synchronic colorectal peritoneal carcinomatosis

**DOI:** 10.7150/jca.46446

**Published:** 2020-07-09

**Authors:** Qian Yan, Zhai Ertao, Zhang Zhimei, Dai Weigang, Peng Jianjun, Chen Jianhui, Chen Chuangqi

**Affiliations:** 1 Division of Gastrointestinal Surgery Center, The First Affiliated Hospital, Sun Yat-sen University, Guangzhou 510080, China.; 2 Gastric Cancer Center, Sun Yat-sen University, Guangzhou 510080, China.; 3 Department of Pathology, The First Affiliated Hospital, Sun Yat-sen University, Guangzhou 510080, China.

**Keywords:** Synchronic colorectal peritoneal carcinomatosis, neutrophil-to-lymphocyte ratio, platelet-to-lymphocyte ratio, systemic immune-inflammation index, prognosis.

## Abstract

**Objective:** Synchronic colorectal peritoneal carcinomatosis (SCRPC) was recognized as a predictor of poor prognosis. The aim of this study was to investigate the role of neutrophil-to-lymphocyte ratio (NLR), platelet-to-lymphocyte ratio (PLR) and systemic immune-inflammation index (SII) on the survival outcome, which might help determine the treatment management of SCRPC patients.

**Methods:** A total of 103 SCRPC patients following cytoreduction surgery (CRS) and systematic chemotherapy (CT) between 1997 and 2013 in the First Affiliated Hospital of Sun Yat-sen University were retrospectively analyzed. The comparison of the clinicopathological variables and systematic inflammatory biomarkers, including NLR, PLR and SII, was performed by Chi-test and Cox regression analysis. According to the results of multivariate analysis, a prognostic nomogram was generated, and its prediction ability was measured by the concordance index (C-index). The survival curves were generated using the Kaplan-Meier method and survival comparison between groups was conducted via the log-rank test.

**Results:** Univariate analysis revealed that elevated NLR, PLR and SII were significantly correlate with worse survival outcome. Only low SII value was recognized as an independent favorable prognostic factor for overall survival (HR=1.772, 95% CI=1.015-3.095, *P*=0.044), except for NLR and PLR. The nomogram could perform well in the prediction of overall survival in SCRPC patients (c-index 0.782). Moreover, SII had strong prognostic discriminatory ability to predict survival outcome for the patients receiving completeness of cytoreduction score (CCR) 0/1 or CCR2/3, rather than NLR and PLR.

**Conclusions:** SII was a better inflammation factor to predict the outcomes of SCRPC patients receiving CRS and systematic CT. Low SII value was the most favorable factor benefiting from different level of CRS and it was useful for determining the appropriate treatment strategy for SCRPC patients.

## Introduction

Approximately 5-10% of colorectal cancer (CRC) patients have synchronic peritoneal metastasis (PM) 1, 2 and PM is the second most frequent CRC metastatic site at the time of initial diagnosis 3. Synchronic colorectal peritoneal carcinomatosis (SCRPC) is regarded as a poor prognostic factor for CRC patients 4. Franko 4 analyzed the database of Analysis and Research in Cancers of the Digestive System (ARCAD) and reported that patients with isolated peritoneal metastasis had worse median survival times (16.3 months) than those with liver (19.1 months), lung (24.6 months), or lymph node (19.4 months) only respectively. Moreover the tumor biological behavior, survival outcome and therapeutic effect of isolated PM origined from CRC significantly differed from other subsets of metastatic colorectal cancer (mCRC) 6, 7. It was important to find out appropriate prognostic markers to predict survival outcome and guide surgeons to choose optimal treatment strategies for SCRPC patients.

Accumulating evidence supported that the interplay between local immune response and systemic inflammation may play a fundamental role in the development and progression of various cancers 8-10, including CRC 11, 12. The levels of neutrophil, lymphocyte and platelet via the complete blood count may shed light on the systemic inflammatory response. However, the inflammatory parameters alone may be easily influenced by other factors, thus the joined tools of inflammatory indices, such as neutrophil to lymphocyte ratio (NLR), platelet to lymphocyte ratio (PLR), may be theoretically more reliable and have potentials as powerful candidates to evaluate the host immune status. Likewise, growing evidence supported a strong close relationship between elevated NLR and PLR and worse survival outcome in CRC patients 13, 14. Systemic immune-inflammation index (SII) was a novel systemic inflammatory index, based on neutrophil, platelet and lymphocyte counts. We previously reported that SII can provided more promising prognostic information than NLR and PLR in CRC patients following radical surgery 15. However, to the best of our knowledge, the prognostic significance of these inflammatory indices, like NLR, PLR and SII, for the SCRPC cases following cytoreduction surgery (CRS) and adjuvant systemic chemotherapy still has not been well studied.

Hence this study aimed to evaluate and compare the prognostic value of different inflammatory indices in SCRPC patients who underwent CRS and systemic chemotherapy and to select an optical inflammatory factor to reflect survival outcome.

## Methods

### Study population

This study was retrospectively analyzed from the database of the Gastrointestinal Surgical Center of the First Affiliated Hospital of Sun Yat-sen University between January 1997 and December 2013. The inclusion criteria were listed below:(1) pathological diagnosis of colorectal adenocarcinoma with PM; (2) patients received cytoreductive surgery and systematic chemotherapy; (3) isolated PM was confirmed according to preoperative findings and intraoperative exploration; (4) patients with complete perioperative clinicopathological, laboratory records and therapeutic interventions. Patients with following criteria were excluded: (1) preoperative anti-tumor or anti-immune treatment, such as chemotherapy, radiotherapy, chemoradiotherapy, immunotherapy; (2) incomplete preoperative clinicopathological and laboratory data or loss of follow-up data; (3) concurrent cancers, recurrent disease or a history of other malignancies within the preceding 5 years; (4) clinical or radiological evidence of inflammatory, infectious or other autoimmune diseases; (5) peritoneal involvement was not confirmed by the pathological examination. At last, 103 SCRPC cases were included in this study. No one had intraoperative chemotherapy or postoperative hyperthermic intraperitoneal chemoperfusion (HIPEC). Patients received postoperative chemotherapy based on a 5-fluorouracil and platinum regimen.Postoperative systemic chemotherapy. The data of the patient's last contact was used as the end of follow-up in all censored patients. Follow-up was updated until December 2018. The study was approved by the independent Ethics Committees of the First Affiliated Hospital of Sun Yat-sen University and was performed in accordance with the ethical standards of the World Medical Association Declaration of Helsinki.

### Data collection

The collected clinical and pathological data in this study was listed as follows: age, gender, preoperative blood test (NLR and PLR), intraoperative blood transfusion, serum carcinoembryonic antigen (CEA), location of primary tumor, histological type, depth of tumor invasion (T stage), lymph node involvement (N stage), extend of PM, completeness of CRS.

All the blood tests were performed within 7 days before surgery. NLR and PLR were calculated as the radio of absolute neutrophils counts and platelet counts divided by absolute lymphocyte counts. SII was calculated using the following formula: SII=P*N/L. where P, N, and L refer to the peripheral platelet, neutrophils, and lymphocyte counts, respectively.

Location of primary tumor was classified into two categories: right-colon and left-colon. Right-colon included cecum, ascending colon and transverse colon. Left-colon included descending colon, sigmoid colon and rectum. T stage and N stage were reclassified according to the 8^th^ edition of TNM classification.

A complete abdominal cavity exploration was performed to evaluate the extent of peritoneal seeding, which was recorded as PCI score 16. Eligible patients were divided into two groups based on the extent of peritoneal carcinomatosis: limited (PCI≤13) and extended (PCI>13).

Completeness of cytoreduction score (CCR) was marked at the end of CRS to assess the volume of residual disease and classified into four categories: CCR0 indicated no macroscopic residual cancer remained; CCR1 indicated no residual nodule larger than 5mm in diameter remained; CCR2 indicated the residual nodule ranged from 5mm to 2.5cm in diameter remained; CCR3 indicated the residual nodule larger than 2.5cm remained. We divided patients into two groups: CCR0/1 and CCR2/3.

### Statistical analysis

All the analyses were conducted using SPSS software 20.0 (SPSS Inc., Chicago, USA). Receiver operating characteristic (ROC) curves with Youden Index correction [maximum (sensitivity+specificity-1)] were used to calculate the optimal prognostic cutoff value of NLR, PLR and SII. Chi-square tests were used to analyze the relationship between NLR, PLR, SII and clinicopathological parameters. The survival curves were drawn using the Kaplan-Meier method and survival comparison between groups was conducted via the log-rank test. Only the variables which were significant prognostic parameters in the univariate Cox's proportional hazards model were included in the multivariate analysis to identify independent prognostic factors for SCRPC patients. The nomogram was explored by the “rms” package of R v3.0.0 software (Institute for Statistics and Mathematics, Vienna, Austria) and the concordance index (C-index) was calculated to predict the performance of the established nomogram model. Statistical significance was established at *p*<0.05 for all tests.

## Results

### ROC curve analysis of inflammatory indices of SCRPC patients

**Figure 1:**
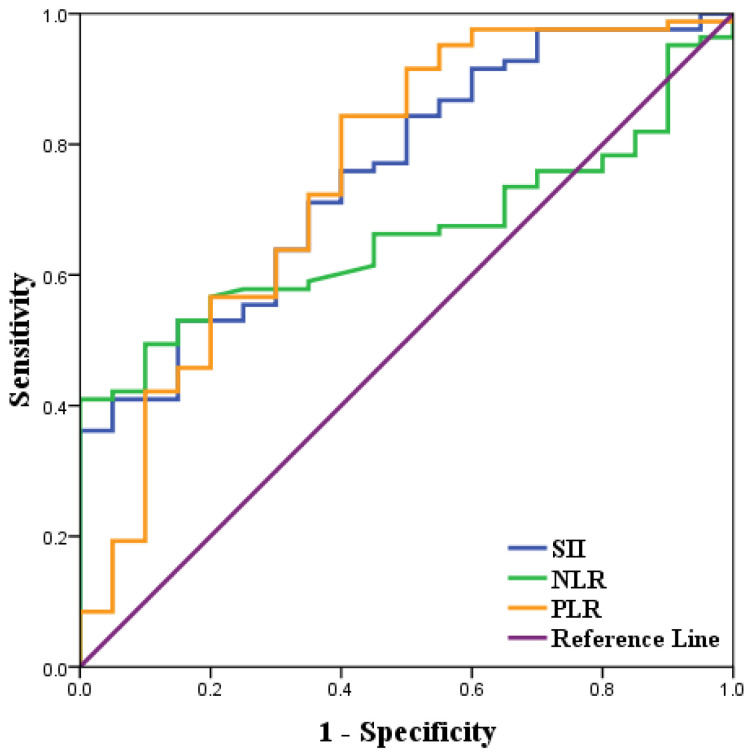
Receiver operating characteristics curve analysis of NLR, PLR, and SII in SCRPC patients following CRS and systemic chemotherapy.

ROC curve analysis was used to determine the best cut-off values of NLR, PLR, SII of 2.6 (AUC=0.662, 95% CI: 55.8%-76.6%, Se=49.4%, Sp=90.0%), 144 (AUC=0.755, 95% CI: 64.5%-86.5%, Se=56.6%, Sp=80.0%), 410 (AUC=0.755, 95% CI: 64.5%-86.5%, Se=75.9%, Sp=60.0%). Among 103 included patients, NLR≥2.6, PLR≥144, SII≥410 were considered as high groups based on the above cut-off results (Fig 1 &Table 1).

### Clinicopathological Characteristics of SCRPC patients

Based on the cut-off results above, included patients were divided into two groups: 62 and 41 had low and high NLR values, 50 and 53 had low and high PLR values, 32 and 71 had low and high SII values. Baseline clinicopathological parameters of SCRPC cases following CRS and systematic chemotherapy were shown in Table 2. There was no correlation between high NLR, PLR, SII value and poor histological type, elevated CEA level, larger tumor size, tumor invasion and lymph node involvement, which all considered as negative prognostic factors for CRC patients. The tumor in the high NLR and SII group inclined to locate in the right-hemicolon, rather than tumor in the high PLR group. Among eligible SCRPC patients, larger proportions of extended peritoneal carcinomatosis were found in high NLR (48.8% vs 14.5%, *p*<0.001), PLR (49.1% vs 6.0%, *p*<0.001) and SII (38.0% vs 6.2%, *p*=0.001) groups respectively. Moreover, in the cases with low NLR, PLR and SII value, the ratio of patients following CCR0/1 surgery was even greater, increasing from 46.3% to 79.0%, 45.3% to 88.0%, 56.3% to 87.5%, respectively.

### Overall survival

The mean follow-up time for this study was 55.4 months. In this cohort, the median OS was 23.8 months, while 2, 5, 10-years overall survival rate was 50.0%, 34.0% and 24.2%, respectively.

**Figure 2:**
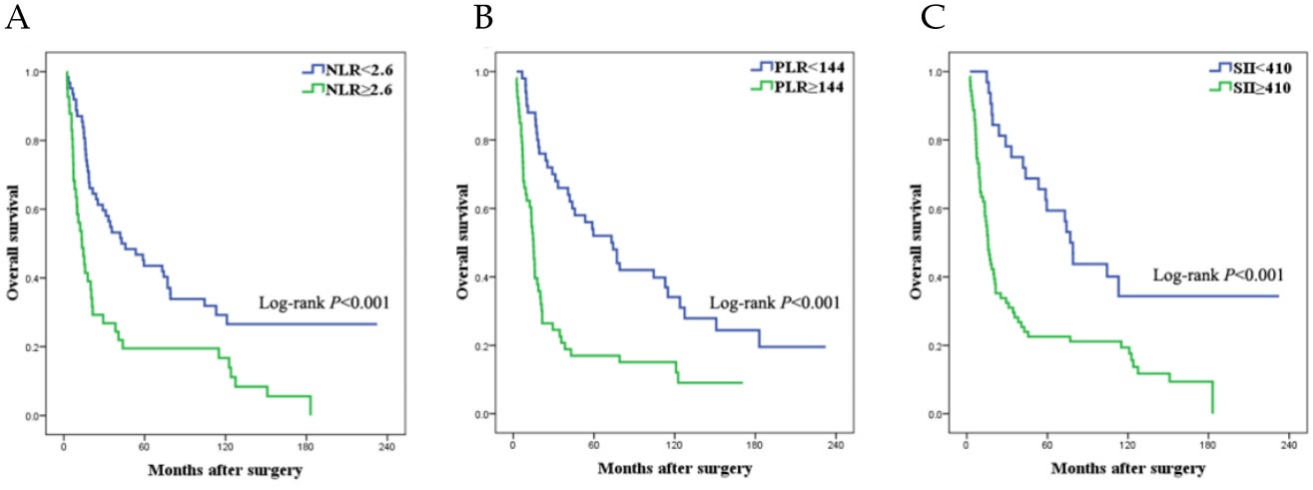
Survival curves of SCRPC patients following CRS and systemic chemotherapy in different NLR (a), PLR (b), and SII (c) group.

The survival data was listed in Table 2. The median survival time of patients in low NLR, PLR and SII group was 44.0, 74.4, 79.2 months and it in high NLR, PLR and SII group was 14.7, 16.1, 17.7 months respectively. Using log-rank test, the low NLR, PLR and SII group statistically had better long-term overall survival outcome than the high NLR, PLR and SII group respectively (Figure 2a-c).

Among all the included factors, univariate analysis showed that age, tumor location, histological type, CEA level, N stage, extent of peritoneal carcinomatosis, completeness of CRS, NLR value, PLR value and SII value were associated with prognosis, whereas age, gender, T stage, intraoperative blood transfusion were not significantly associated with OS (Table 3). According to the multivariate analysis, only tumor located in the right-hemicolon, elevated CEA level, extended PM, CCR0/1 and high SII value were independent factors associated with a worse prognosis (Table 3), rather than NRL value and PRL value.

**Figure 3:**
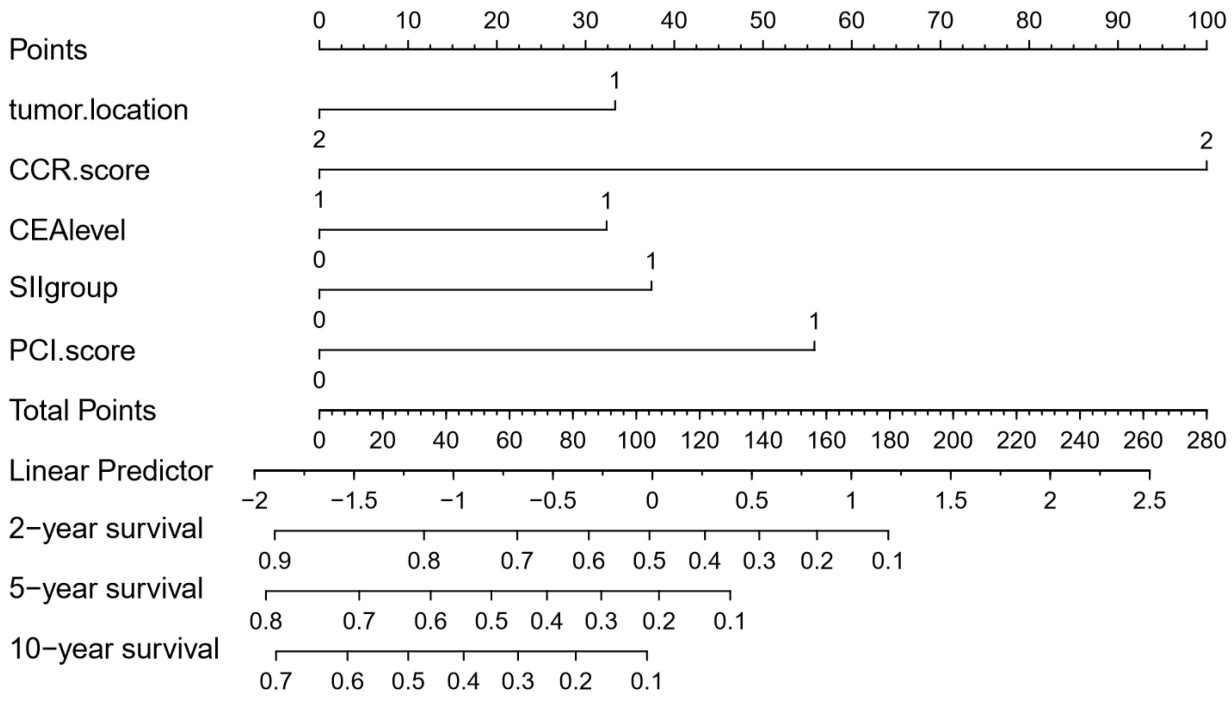
Nomogram of predicting 2-, 5- and 10-year survival for SCRPC patients after CRS and systematic CT. Tumor location: 1, right semicolon; 2, left semicolon; CCR score: 1, CCR0/1; 2, CCR2/3; CEA level: 0, ≦5ng/ml; 1, >5ng/ml; SII group: 0, SII<410; 1, SII≥410; PM level: 0, limited; 1, extended.

To further predict the survival of SCRPC patients after CRS and systematic CT, all the significant independent risk prognostic factors of the primary cohort were integrated in the nomogram to predict the 2-, 5- and 10-year survival using multivariate Cox regression model analysis (Fig. 3). The C-index for OS prediction was 0.782, which closed corresponded to the actual survival.

### Overall survival stratified by completeness of CRS

**Figure 4:**
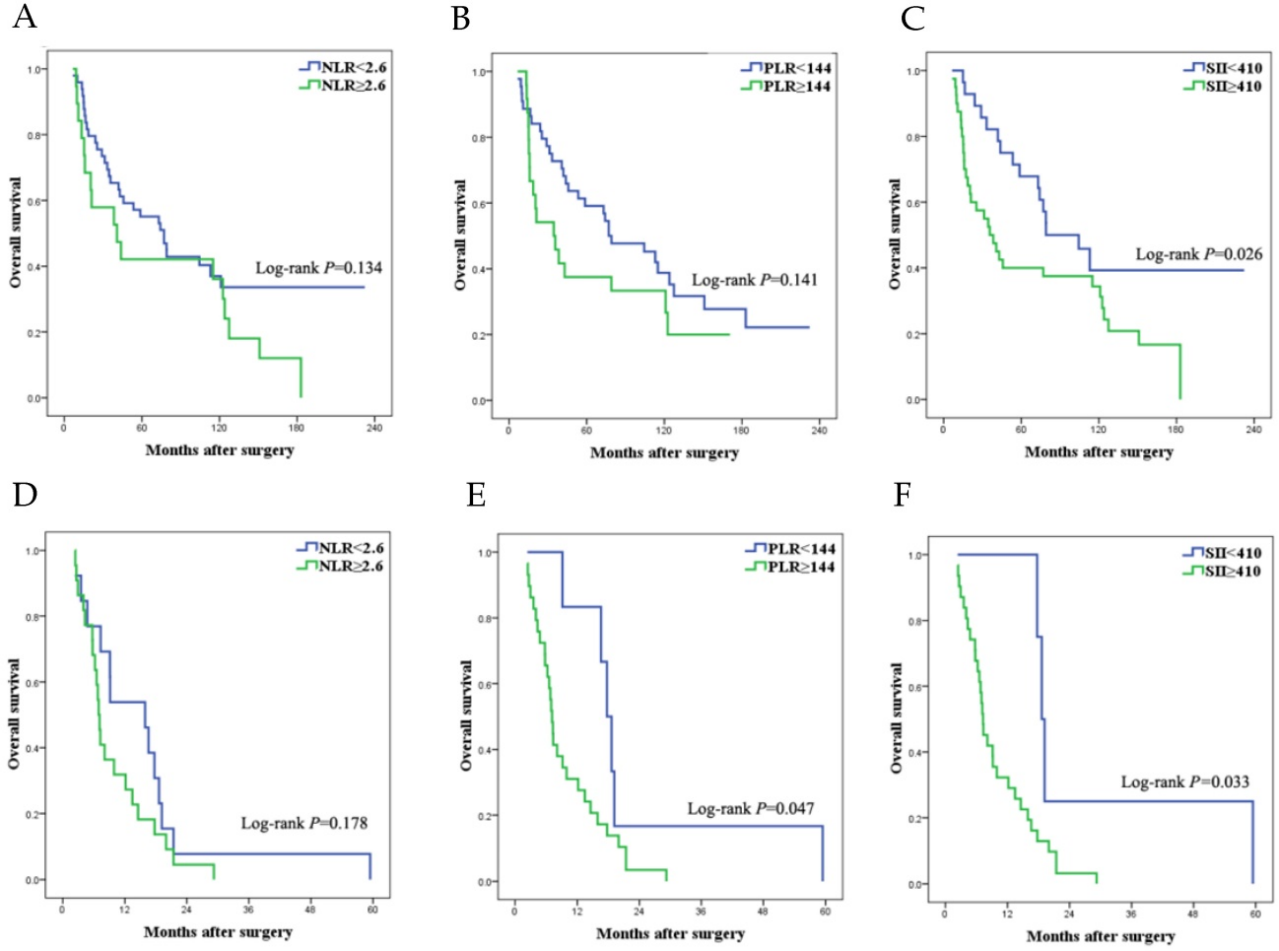
Survival curves of SCRPC patients following different levels of CRS. (a-c) SCRPC patients following CCR 0/1 surgery. a. high vs low NLR group; b. high vs low PLR group; c. high vs low SII group. (d-f) SCRPC patients following CCR 2/3 surgery. d. high vs low NLR group; e high vs low PLR group; f. high vs low SII group.

Among the patients following CCR 0/1 surgery, age, tumor size, T stage, PM level, intraoperative blood transfusion and SII value were associated with overall survival (Table 4). Of these factors, multivariate analysis still showed that low SII value remained a strong prognostic factor for improved overall survival, not NLR and PLR. Survival curve among different levels of NLR, PLR and SII was shown in Figure 4a-c and survival data was listed in Table 2.

Of the 35 (34.0%) patients underwent CCR 2/3 surgery, the survival curves (Fig. 4d-f) were drawn to describe the relationship between OS and NLR, PLR and SII value. Following prognostic factors were significant in univariate analysis: tumor location, CEA level, PM level, PLR value and SII value. Multivariate analysis was further performed for significant factors in univariate test. Only low CEA level and low SII value were regarded as favorable prognostic factor in this cohort (Table 5).

## Discussion

To the best of our knowledge, this was the first retrospective study to analyze the prognostic significance of systematic inflammatory biomarkers in CRC patients with PM. Our results indicated that preoperative NLR, PLR and SII value all had the abilities to predict survival outcome in our cohort and SII was the only independent prognostic factor. Moreover, SII can distinguish the survival differences in the SCRPC cases independently of the completeness level of CRS.

Although significant progresses have been made in terms of developing non-invasive prognostic biomarkers for CRC, the clinical appliance of the systematic inflammatory indices were regularly used, with its advantage of inexpensive, reliable, reproducible, robust and convenience 17-19. Among all the systematic inflammatory indices, NLR and PLR were the most commonly used as prognostic biomarkers in CRC, which even had a discriminatory ability superior to other inflammatory biomarkers in resectable CRC 20. While, SII was only recently introduced. Growing evidence demonstrated that SII has shown optical prognostic power in a variety of malignancies, including bladder cancer 21, non-small cell lung cancer 22, gastric 23, pancreatic cancer 24, and CRC 15. However, no previous studies have investigated the clinical significance of these systematic inflammatory biomarkers in CRC with peritoneal metastasis. Thus, we were the first to reveal that SCRPC patients following CRS and systematic chemotherapy with elevated NLR or PLR or SII levels at initial diagnosis had poor survival outcome. What's more important, SII was the only independent systematic inflammatory prognostic factor to predict long-term survival outcomes of SCRPC patients, not NLR or PLR, which was similar to in primary CRC patients.

Our study showed that the systematic inflammation biomarkers including NLR, PLR and SII correlated with the survival outcome of SCRPC patients. Although precise prognostic mechanism was not completely understood, strong evidences considered that the systematic inflammation may play a critical role in pathogenesis and progression of cancer and predict worse survival outcome, including CRC 25-27. Neutrophils may produce and secret cytokines, chemokines, and proteases, which promoted cancer cells adhesion and seeding in the peritoneum. Lymphopenia destroyed tumor immune defense by inhibiting cancer cells immune surveillance and blocking cytotoxic cell death, which may produce a favorable tumor microenvironment in the peritoneum for the proliferation, progression and spread of CRC cells 28, 29. Recent evidence suggested that platelets can release platelet-derived growth factors and pro-angiogenic protein (such as interleukin-6) and act as chemoattractants to promote growth, migration and angiogensis of tumor cells. Moreover, tumor cells can directly interact with platelets to facilitate tumor extravasation and metastatic niche formation 30-32. As a result, the level of serum platelets was positive correlated with the degrees of inflammatory response. Therefore, the elevated level of neutrophils and platelets can reflect systematic inflammation response and the decline of lymphocytes may be responsible for cellular immune injury or systematic immune surveillance. Hence SII based on the above systematic inflammatory indices can better and more objectively reflect the balance between host inflammation and immune response in cancer patients, which might make SII a better biomaker of predicting survival outcome in SCRPC patients.

Although overall survival of SCRPC patients has increased over time with the development of multidisciplinary management (such as HIPEC, CRS, IPC, chemotherapy) and new target drugs (such as bevacuzimab, cetuximab), the efficacy of treatment for SCRPC was still yet not established. As Prodige 7 trial 33 failed to demonstrate the survival benefit was gained from HIPEC with oxaliplatin to a complete CRS. Most SCRPC patients were accompanied by synchronous hepatic or lung metastases. SCRPC occupied less than 5% of stage IV CRC cases and had a great tendency to develop metachronous distant metastasis. Hence SCRPC may get a potentially survival improvement from systematic chemotherapy. However, the therapeutic concentration in the peritoneal metastatic nodule following systemic administration of chemotherapy was relatively low, possibly because of the lack of vascularization of PC seeding. Thus, some researchers proposed that SCRPC patients without extraperitoneal disease may get more survival benefit from locoregional treatment, such as complete cytoreduction (Ref??). The median survival time of SCRPC patients who underwent CRS was more than 30 months and 5 year survival rate was nearly 30% 34, similar to the Stage III CRC patients, but, not all patients got survival benefit from complete cytoreduction. The prognostic value of these treatment was still controversial. Therefore, better and more powerful prognostic factors should be explored to help selecting patients who can benefit of such extensive procedure. The Peritoneal Surface Disease Severity Score (PSDSS) 35 and colorectal peritoneal metastases prognostic surgical score (COMPASS) 36 were reported to had a good discriminative ability to improve patient selection. In this study we firstly focused on the clinical appliances of systematic inflammatory factors to predict survival in these patients. The result in our cohort demonstrated that SII was the only systematic inflammatory index who had a stronger prognostic ability to discriminate survival differences independent of the levels of complete cytoreduction and systematic chemotherapy.

There were still some limitations in this study. First, this was a single-center study and the study population was small, so more patients from other centers will be needed for further external validation. Second, although the systematic inflammatory indices were recorded prospectively, the analysis of this study was undertaken in a retrospective fashion. Also, the study was only focused on the OS of selected SCRPC patients who underwent CRS and systematic chemotherapy, and more prognostic indicator will be included in further studies.

## Conclusions

This was the first report to demonstrate that SII had a stronger prognostic value among SCRPC patients, and SII can provide prognostic value to distinguish survival outcome independently of the level of CRS, rather than NLR and PLR. SII was recommended as the prognostic factors to select appropriate SCRPC patients who can benefit from CRS and systematic chemotherapy. However, prospective studies and the larger number of patients are needed to further validate this finding.

## Figures and Tables

**Table 1:** Baseline SCRPC patient characteristics based on.

	Cases	NLR		*P* value	PLR		*P* value	SII		*P* value
		NLR<2.6	NLR≥2.6		PLR<144	PLR≥144		SII<410	SII≥410	
Age(years)				0.183			0.131			0.266
≦60	56	37(59.7%)	19(46.3%)		31(62.0%)	25(47.2%)		20(62.5%)	36(50.7%)	
>60	47	25(40.3%)	22(53.7%)		19(38.0%)	28(52.8%)		12(37.5%)	35(49.3%)	
Gender				0.260			0.151			0.934
Male	67	43(69.4%)	24(58.5%)		36(72.0%)	31(58.5%)		21(65.6%)	46(64.8%)	
Female	36	19(30.6%)	17(41.5%)		14(28.0%)	22(41.5%)		11(34.4%)	25(35.2%)	
Tumor location				<0.001			0.110			0.007
Right-sided	39	15(24.2%)	24(58.5%)		15(30.0%)	24(45.3%)		6(18.8%)	33(46.5%)	
Left-sided	64	47(75.8%)	17(41.5%)		35(70.0%)	29(54.7%)		26(81.2%)	38(53.5%)	
Histological type				0.068			0.347			0.250
Well-differentiated	59	40(64.5%)	19(46.3%)		31(62.0%)	28(52.8%)		21(65.6%)	38(53.5%)	
Poor-differentiated	44	22(35.5%)	22(53.7%)		19(38.0%)	25(47.2%)		11(34.4%)	33(46.5%)	
CEA level				0.544			0.632			0.449
≦5ng/ml	49	31(50.0%)	18(43.9%)		25(50.0%)	24(45.3%)		17(53.1%)	32(45.1%)	
>5ng/ml	54	31(50.0%)	23(56.1%)		25(50.0%)	29(54.7%)		15(46.9%)	39(54.9%)	
Tumor size				0.192			0.554			0.159
≦5cm	38	26(41.9%)	12(29.3%)		17(34.0%)	21(39.6%)		15(46.9%)	23(32.4%)	
>5cm	65	36(58.1%)	29(70.7%)		33(66.0%)	32(60.4%)		17(53.1%)	48(67.6%)	
Intraoperative blood transfusion				0.304			0.705			0.959
No	39	21(33.9%)	18(43.9%)		18(36.0%)	21(39.6%)		12(37.5%)	27(38.0%)	
Yes	64	41(66.1%)	23(56.1%)		32(64.0%)	32(60.4%)		20(62.5%)	44(62.0%)	
T stage				0.958			0.317			0.598
T3	38	23(37.1%)	15(36.6%)		16(32.0%)	22(41.5%)		13(40.6%)	25(35.2%)	
T4	65	39(62.9%)	26(63.4%)		34(68.0%)	31(58.5%)		19(59.4%)	46(64.8%)	
Lymph node metastasis				0.677			0.691			0.462
N^0^	17	11(17.7%)	6(14.6%)		9(18.0%)	8(15.1%)		4(12.5%)	13(18.3%)	
N^+^	86	51(82.3%)	35(85.4%)		41(82.0%)	45(84.9%)		28(87.5%)	58(81.7%)	
PM level				0.001			<0.001			0.001
limited	74	52(83.9%)	22(53.7%)		47(94.0%)	27(50.9%)		30(93.8%)	44(62.0%)	
extended	29	10(16.1%)	19(46.3%)		3(6.0%)	26(49.1%)		2(6.2%)	27(38.0%)	
CCR score				0.001			<0.001			0.002
CCR0/1	68	49(79.0%)	19(46.3%)		44(88.0%)	24(45.3%)		28(87.5%)	40(56.3%)	
CCR2/3	35	13(21.0%)	22(53.7%)		6(12.0%)	29(54.7%)		4(12.5%)	31(43.7%)	

**Table 2:** Survival rate of OS for SCRPC patients.

	Low value			High value			*P* value
	2ysr	5ysr	10ysr	2ysr	5ysr	10ysr	
Whole							
NLR	62.9%	43.5%	29.3%	29.3%	19.5%	16.7%	<0.001
PLR	74.0%	52.0%	34.2%	26.4%	17.0%	15.1%	<0.001
SII	81.3%	59.4%	34.6%	35.2%	22.5%	19.4%	<0.001
CCR0/1							
NLR	77.6%	55.1%	37.0%	57.9%	42.1%	36.1%	0.134
PLR	81.8%	59.1%	38.9%	54.2%	37.5%	33.3%	0.141
SII	60.0%	40.0%	34.4%	89.3%	67.9%	39.6%	0.026
CCR2/3							
NLR	7.7%	0.0%	0.0%	4.5%	0.0%	0.0%	0.178
PLR	16.7%	0.0%	0.0%	3.4%	0.0%	0.0%	0.047
SII	25.0%	0.0%	0.0%	3.2%	0.0%	0.0%	0.033

**Table 3:** Univariate and multivariate analyses of factors affecting CRC patients with peritoneal metastases after palliative primary tumor resection and cytoreductive surgery.

	Univariate analysis				Multivariate analysis			
	χ^2^ value	HR	95%CI	*P* value	χ^2^ value	HR	95%CI	*P* value
Age (≦60y vs >60y)	3.575	-	-	0.059				
Gender (male vs female)	0.712	-	-	0.399				
Tumor location (left vs right)	4.615	0.619	0.400-0.959	0.032	4.019	0.618	0.385-0.989	0.045
Histological type (well vs poor-differentiated)	3.957	1.551	1.006-2.390	0.047				
Tumor size (≦5cm vs >5cm)	0.801	-	-	0.371				
CEA level (≦5ng/ml vs >5ng/ml)	4.661	1.614	1.045-2.493	0.031	6.959	1.844	1.170-2.904	0.008
T stage (T_3_ vs T_4_)	1.551	-	-	0.213				
Lymph node metastasis (N_0_ vs N_+_)	4.487	1.995	1.053-3.781	0.034				
PM level (limited vs extended)	46.668	3.684	2.534-5.356	<0.001	4.530	2.340	1.070-5.118	0.033
Completeness of CRS (CRS0/1 vs CRS2/3)	53.634	6.982	4.151-11.745	<0.001	15.347	4.471	2.114-9.459	<0.001
Intraoperative blood transfusion (no vs yes)	3.571	-	-	0.059				
NLR value (low vs high)	12.241	2.174	1.407-3.359	<0.001				
PLR value (low vs high)	17.535	2.593	1.660-4.050	<0.001				
SII value (low vs high)	14.057	2.651	1.592-4.413	<0.001	4.048	1.772	1.015-3.095	0.044

**Table 4:** Univariate and multivariate analysis of factors affecting SCRPC patients following CCR0/1.

	Univariate analysis				Multivariate analysis			
	χ^2^ value	HR	95%CI	*P* value	χ^2^ value	HR	95%CI	*P* value
Age (≦60y vs >60y)	3.892	1.784	1.004-3.171	0.049	8.087	2.473	1.325-4.614	0.004
Gender (male vs female)	0.415	-	-	0.519				
Tumor location (left vs right)	2.967	-	-	0.085				
Histological type (well vs poor-differentiated)	2.387	-	-	0.122				
Tumor size (≦5cm vs >5cm)	4.570	0.531	0.297-0.949	0.033				
CEA level (≦5ng/ml vs >5ng/ml)	1.743	-	-	0.187				
T stage (T_3_ vs T_4_)	6.805	0.461	0.258-0.825	0.009	6.287	0.463	0.254-0.845	0.012
Lymph node metastasis (N_0_ vs N_+_)	3.141	-	-	0.076				
PM level (limited vs extended)	9.475	6.956	2.023-23.917	0.002	15.190	14.081	3.724-53.240	<0.001
Intraoperative blood transfusion (no vs yes)	5.879	0.485	0.270-0.871	0.015	5.864	0.470	0.255-0.866	0.015
NLR value (low vs high)	2.246	-	-	0.134				
PLR value (low vs high)	2.164	-	-	0.141				
SII value (low vs high)	4.753	1.963	1.071-3.601	0.029	6.502	2.277	1.210-4.288	0.011

**Table 5:** Univariate and multivariate analysis of factors affecting SCRPC patients following CCR2/3.

	Univariate analysis				Multivariate analysis			
	χ^2^ value	HR	95%CI	*P* value	χ^2^ value	HR	95%CI	*P* value
Age (≦60y vs >60y)	0.136	-	-	0.713				
Gender (male vs female)	0.661	-	-	0.416				
Tumor location (left vs right)	4.611	0.446	0.213-0.932	0.032				
Histological type (well vs poor-differentiated)	1.755	-	-	0.185				
Tumor size (≦5cm vs >5cm)	2.314	-	-	0.128				
CEA level (≦5ng/ml vs >5ng/ml)	5.342	2.307	1.135-4.687	0.021	6.123	2.484	1.208-5.107	0.013
T stage (T_3_ vs T_4_)	0.166	-	-	0.684				
Lymph node metastasis (N_0_ vs N_+_)	1.154	-	-	0.283				
PM level (limited vs extended)	4.758	2.456	1.095-5.505	0.029				
Intraoperative blood transfusion (no vs yes)	1.187	-	-	0.276				
NLR value (low vs high)	1.1779	-	-	0.182				
PLR value (low vs high)	3.626	2.547	0.973-6.668	0.057				
SII value (low vs high)	3.952	3.388	1.017-11.287	0.047	4.495	3.721	1.104-12.535	0.034
